# Beyond the Package Insert: A Postmarket Digital Ethnographic Study of FDA-Cleared Prescription Digital Therapeutics

**DOI:** 10.7759/cureus.84450

**Published:** 2025-05-20

**Authors:** Shaheen E Lakhan

**Affiliations:** 1 Medical Office, Click Therapeutics, Inc., New York, USA; 2 Department of Neurology, Western University of Health Sciences, Pomona, USA; 3 Department of Neurology, A.T. Still University School of Osteopathic Medicine in Arizona, Mesa, USA; 4 Department of Neurology, Morehouse School of Medicine, Atlanta, USA; 5 Department of Bioscience, Global Neuroscience Initiative Foundation, Miami, USA

**Keywords:** digital ethnography, digital health, emotional impact, engagement, fda-cleared, postmarket surveillance, prescription digital therapeutics, reddit, usability, user experience

## Abstract

Introduction

Prescription digital therapeutics (PDTs) are FDA-cleared software interventions designed to treat a variety of clinical conditions. Despite clinical validation through randomized controlled trials, PDTs face postmarket challenges that remain unmonitored through traditional surveillance. This study introduces a novel framework of digital ethnography, utilizing social media, specifically Reddit, to capture spontaneous, community-driven insights on PDT experiences beyond clinical trials. It seeks to explore the nuanced realities of user engagement, interface friction, emotional impact, and public perception.

Methods

We conducted a digital ethnographic analysis of Reddit posts collected from condition-specific and general health subreddits. We focused on 13 FDA-cleared PDTs. Posts containing first-person accounts or caregiver perspectives were included, and a thematic analysis was performed to identify recurrent themes related to user experience. Descriptive statistics were generated to support qualitative findings.

Results

The study revealed that Reddit communities offer rich, culturally-specific contexts that shape PDT experiences. A final sample of 65 posts, each containing first-person or caregiver perspectives, was analyzed, discussing 11 of the 13 PDTs. Five major domains emerging included ambivalence about efficacy (belief and skepticism), common patterns of engagement and dropout (motivation and frustration), usability challenges (technical friction), and structural barriers to access (insurance and affordability). Additionally, users frequently expressed emotional meaning-making as they navigated their therapeutic journeys. Differences in discussion tone were noted across subreddits: for example, r/ADHD focused on pragmatic usability, while r/depression engaged in deeper emotional reflection.

Discussion

This digital ethnographic study highlights the importance of integrating user-generated data from digital platforms like Reddit to understand the real-world impact of PDTs. While clinical trials offer limited insights, social media provides a broader, more diverse range of experiences that reveal common usability barriers, engagement patterns, and access challenges. Our findings suggest that the context in which PDTs are discussed, shaped by community norms, beliefs, and expectations, plays a critical role in shaping user outcomes and perceptions.

Conclusion

Postmarket digital ethnography offers valuable insights into the user experience of FDA-cleared PDTs, which are not captured in traditional clinical trials or regulatory processes. This study advocates for the inclusion of structured social media analysis as part of postmarket surveillance to improve the design, accessibility, and effectiveness of PDTs. Listening to the voice of the patient in informal spaces like Reddit is essential for ensuring the real-world success of digital therapeutics.

## Introduction

Prescription digital therapeutics (PDTs) represent an emerging category of medical software interventions, formally reviewed by the FDA for the treatment of specific clinical conditions [1]. These include neurological, psychiatric, gastrointestinal, and metabolic disorders, and the products themselves range from digital cognitive behavioral therapy to neuromodulation. While clinical validation of PDTs is grounded in randomized controlled trials, these trials provide safety and efficacy data under controlled conditions but often fail to capture the complexity of real-world use, including user engagement, emotional response, and practical usability challenges. Factors such as technical reliability, interface design, and onboarding processes can significantly influence whether users stay engaged and complete treatment, especially when using PDTs without professional support.

Traditionally, the safety and effectiveness of medical device products are evaluated post-approval through structured mechanisms such as the FDA's Manufacturer and User Facility Device Experience (MAUDE) database, insurance claims data, and clinician-reported outcomes [2]. However, these sources offer limited insight into the spontaneous, community-mediated experiences, those shaped not only by individual users but also by the cultural norms, shared language, and expectations of online communities, that influence how patients and caregivers actually engage with PDTs. Increasingly, online platforms such as Reddit (Reddit Inc., San Francisco, California, United States) serve as informal yet deeply informative repositories of organically generated, user-initiated narratives reflecting real-world therapeutic journeys [3].

This study proposes a novel framework: the digital ethnography of PDTs [4]. Drawing on naturalistic posts and discussions across Reddit, we aim to characterize the sociotechnical experience of PDTs as it unfolds in everyday life. This includes not only adverse events and usability barriers but also themes of empowerment, skepticism, dropout, and emotional meaning-making. Specifically, we asked: How do Reddit users describe their experiences with PDTs in terms of usability, engagement, and emotional impact? What themes emerge across different subreddit communities that reflect real-world therapeutic experiences? The result is a multidimensional view of how PDTs are received, enacted, and interpreted beyond the boundaries of clinical trials.

## Materials and methods

We conducted a digital ethnographic analysis of Reddit posts and comments mentioning FDA-cleared PDTs between January 1, 2019, and May 4, 2025 [5]. Data were collected using Apify (Apify Technologies s.r.o., Prague, Czech Republic), a cloud-based web scraping platform used to automate the extraction of publicly available posts. We used a custom query set designed to retrieve references to 13 publicly known FDA-cleared PDTs: EndeavorRx (Akili Interactive, Boston, Massachusetts, United States) [6], reSET (PursueCare, Middletown, Connecticut, United States) [7], reSET-O (PursueCare, Middletown, Connecticut, United States) [8], Somryst (Nox Health, Alpharetta, Georgia, United States) [9], Parallel (Mahana Therapeutics, San Francisco, California, United States) [10], Regulora (metaMe Health, Chicago, Illinois, United States) [11], Rejoyn (Otsuka Precision Health, Princeton, New Jersey, United States) [12], CT-132 (Click Therapeutics, New York, New York, United States) [13], Stanza (Swing Therapeutics, San Francisco, California, United States) [14], MamaLift Plus (Curio Digital Therapeutics, Princeton, New Jersey, United States) [15], SleepioRx (Big Health, San Francisco, California, United States) [16], DaylightRx (Big Health, San Francisco, California, United States) [17], and AspyreRx (Click Therapeutics, New York, New York, United States) [18]. Each product was queried using multiple keyword permutations, including brand, indication, and manufacturer where necessary. For example, posts related to EndeavorRx were searched using the terms "EndeavorRx", "EndeavorRx ADHD", and "Akili Interactive". Broader terms such as "digital therapeutic", "prescription digital therapeutic", and "PDT" were also included to capture relevant posts discussing the digital therapeutic category more generally.

Posts were collected across condition-relevant and general health subreddits, including r/ADHD, r/depression, r/digitalhealth, r/mentalhealth, r/insomnia, r/migraine, and r/chronicpain. Results were exported to CSV format and manually reviewed for inclusion. Eligible posts contained first-person accounts or caregiver perspectives describing use, experience, impressions, or outcomes related to PDTs. Non-English, off-topic, promotional, or duplicate content was excluded.

An inductive thematic analysis was performed, with codes emerging from repeated close reading of the dataset. These codes were clustered into higher-order categories representing the ethnographic domains of user belief, engagement, usability, access, and emotional meaning-making [19]. Representative quotes were extracted, anonymized, and used to illustrate key themes in the Results section. Basic descriptive statistics were generated to support qualitative findings, including frequency distributions by theme and illustrative subreddits. As the thematic analysis was conducted by a single author, inter-coder reliability was not applicable.

Ethical considerations

While Reddit data is publicly available, the analysis of health-related discussions requires careful attention to ethical principles. To ensure privacy and confidentiality, all posts were anonymized by removing usernames. To further reduce the risk of re-identification, all quotes were paraphrased using a meaning-preserving approach that alters wording and phrasing while maintaining the original sentiment and context. This process follows best practices in digital ethnography to ensure that individual posts cannot be traced via direct search. Given the public and anonymized nature of the data, the study did not require Institutional Review Board (IRB) approval, as per standard practice for the analysis of publicly available, de-identified online data. This exemption ensures that the ethical guidelines surrounding the use of digital ethnography are respected while still maintaining the credibility and integrity of the research.

## Results

Dataset overview

A total of 751 Reddit posts and comments were collected using Apify between January 2019 and May 2025 (Table 1). All entries contained non-empty content and unique identifiers. Of these, 657 posts were excluded because they did not reference any of the 13 targeted FDA-cleared PDTs. This initial filter yielded 94 potentially relevant posts based on the presence of PDT-related terms in the body text. These posts were then manually reviewed for topical relevance, clarity, and depth of user experience. Specifically, 29 posts were excluded for the following reasons: some discussed unrelated topics or referenced PDTs only in passing without describing personal experiences; others were too brief or vague to yield meaningful thematic data; several were promotional in nature, such as advertisements or manufacturer-sponsored content; and duplicates were removed to ensure analytic uniqueness. A final sample of 65 posts was selected based on experiential richness and relevance, consistent with qualitative research practices that emphasize thematic depth over volume. These posts referenced 11 of the 13 targeted PDTs, including EndeavorRx, reSET, reSET-O, Somryst, Parallel, Regulora, Rejoyn, CT-132, MamaLift Plus, SleepioRx, and AspyreRx. The two remaining PDTs, Stanza and DaylightRx, were excluded due to a lack of relevant posts meeting the inclusion criteria.

**Table 1:** Data filtering flow summary This author-created table summarizes the filtering process from initial Reddit data collection to the final analytic sample used for qualitative coding. Of 751 total posts, 94 referenced at least one of the 13 FDA-cleared PDTs targeted. After manual review, 29 posts were excluded for lacking sufficient experiential content, resulting in a final sample of 65 posts analyzed for thematic content. PDTs: prescription digital therapeutics

Stage	Description	Count
Initial posts collected	Reddit posts and comments containing non-empty content, collected using Apify between January 2019 and May 2025	751
Posts referencing PDTs	Identified using keyword permutations related to 13 FDA-cleared PDTs	94
Posts excluded after manual review	Excluded for being off-topic, promotional, or duplicates or lacking first-person experience	29
Final posts included in the thematic analysis	Posts selected based on depth, clarity, and relevance	65

Subreddit cultures and therapeutic discourse

Reddit subreddits function as cultural microenvironments, each with distinct norms, expectations, and communicative tones [20]. Analysis revealed that the context in which a PDT was discussed had a profound influence on the framing and content of user experiences. While some subreddits emphasized clinical outcomes and treatment skepticism, others foregrounded emotional support, pragmatic usability, or community consensus (Table 2).

**Table 2:** Subreddit cultures and therapeutic discourse This author-created table summarizes the dominant tone, commonly discussed PDTs, and key experiential themes within each subreddit included in the analysis. Each subreddit functioned as a unique sociocultural environment that shaped how users discussed and interpreted their experiences with the PDT. PDTs: prescription digital therapeutics

Subreddit	Predominant tone	Commonly mentioned PDTs	Key themes highlighted
r/ADHD	Parental, pragmatic	EndeavorRx	Daily use, screen time concerns, optimism
r/depression	Introspective, emotional	Rejoyn, reSET-O	Mental health identity, doubt, personal journey
r/digitalhealth	Analytical, commercial	Somryst, Regulora	Regulatory trends, industry, credibility
r/insomnia	Frustrated, functional	Somryst, SleepioRx	Access barriers, onboarding confusion
r/migraine	Uncertain, exploratory	CT-132	App confusion, symptom overlap, curiosity

In r/ADHD, EndeavorRx was often discussed by parents or guardians managing pediatric treatment. Posts reflected both hope and doubt, with several caregivers expressing optimism about digital alternatives to medication, while others voiced concerns about screen time, attention reinforcement, or the app's novelty wearing off. The tone was generally practical, focused on day-to-day integration and compliance.

By contrast, r/depression featured more introspective and emotionally charged accounts, especially surrounding Rejoyn. Users in this subreddit often disclosed personal struggles, articulating both positive expectations and deep skepticism about whether a phone-based intervention could meaningfully impact mood. Posts frequently blended digital therapeutic discussion with broader narratives of mental health journeying, loneliness, and identity.

The r/digitalhealth subreddit exhibited a markedly different tone, one that is professional, analytical, and innovation-driven. Here, PDTs were discussed in terms of regulatory milestones, commercialization, and digital health investment. While less emotive, posts in this subreddit provided contextual depth, often linking to news articles, FDA press releases, or investor announcements. Products like Somryst and Regulora appeared as case studies in software-based therapeutics.

Other communities, such as r/insomnia and r/migraine, tended to focus on functional outcomes and frustration with access. Discussions around Somryst often referenced insurance hurdles, onboarding complexity, or mixed results with sleep tracking. In r/migraine, CT-132 was rarely mentioned by name, likely given only very recent clearance.

Taken together, these subreddit cultures illuminate how therapeutic software is interpreted through the lens of community identity, need, and norms. PDTs are not experienced in isolation but within the frameworks of parental responsibility, emotional vulnerability, tech-savviness, or healthcare access frustration. This cultural context fundamentally shapes how users narrate success, failure, or indifference to their digital treatments.

Thematic domains of experience

**Figure 1:**
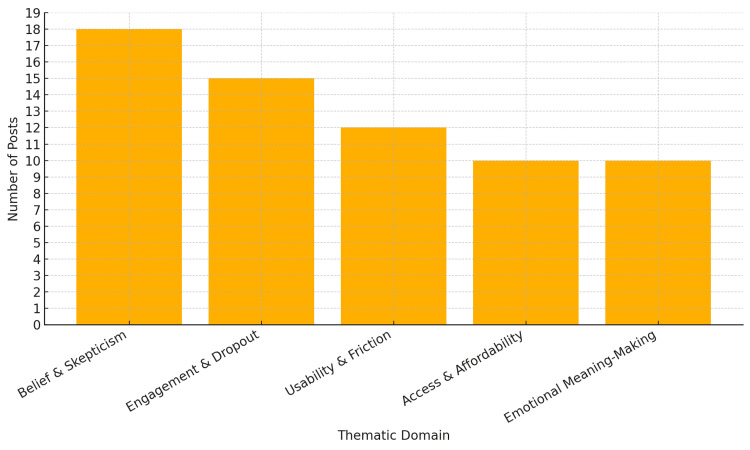
Frequency of thematic domains in Reddit posts about PDTs This author-created bar chart displays the number of Reddit posts coded to each of the five thematic domains identified through inductive thematic analysis of 65 user-generated posts discussing FDA-cleared PDTs. The chart illustrates the relative prominence of key experiential categories: belief and skepticism, engagement and dropout, usability and friction, access and affordability, and emotional meaning-making. Thematic coding was performed iteratively based on close reading of eligible posts, with each post potentially contributing to multiple domains. Notably, usability and engagement challenges emerged as the most frequently reported experiences, underscoring the practical and emotional complexities of PDT use in real-world settings. PDTs: prescription digital therapeutics

In addition to community-specific framing, thematic coding of posts revealed five dominant domains of user experience across products and subreddits [21]: belief and skepticism, engagement and dropout, usability and friction, access and affordability, and emotional meaning-making (Figure 1 and Table 3). These themes emerged inductively from recurrent patterns in how users described their interactions with PDTs and the language they employed.

**Table 3:** Thematic domains with representative Reddit quotes This author-created table presents the main themes identified in Reddit discussions of PDTs, illustrating user experiences, challenges, and perceptions. To protect user anonymity and prevent searchability, all quotes have been paraphrased while preserving their original meaning and contextual nuance, in accordance with ethical standards in digital ethnographic research. PDTs: prescription digital therapeutics

Theme	Description	Example paraphrased quote
Belief and skepticism	Tension between trust in FDA clearance and doubts about efficacy	"Some people assume that long periods of gaming mean a person doesn't really have ADHD, but in reality, game-based tools can actually align well with how ADHD brains function"
Engagement and dropout	Patterns of use and discontinuation	"It seems harder to stay motivated with an app than with a real person"
Usability and friction	Technical challenges in app launch or use	"I struggled to get the app running, and after trying the code a few times without success, I gave up"
Access and affordability	Financial and insurance-related barriers	"It helped while I had coverage, but once the insurance dropped it, I couldn't pay on my own and had to quit"
Emotional meaning-making	Using PDTs to construct therapeutic identity or regain control	"Using the app helped me tie my feelings to actions, something that regular therapy hadn't managed. It felt more than just a game, it felt genuine"

Belief and skepticism often coexisted in the same post. Many users described initial excitement about the potential of a digital therapeutic, particularly when grounded in neuroscience or FDA clearance, but expressed doubt about whether app-based interventions could produce lasting clinical change. This ambivalence was especially prominent in posts about Rejoyn and reSET-O, with users weighing their hopes against previous disappointments with traditional and digital treatments alike.

Engagement and dropout formed a second major theme. Posts included reports of sustained daily use, gamification appeal, and therapeutic value, but also highlighted common patterns of discontinuation. Reasons for disengagement ranged from boredom and repetition to emotional fatigue and perceived inefficacy. Several users discussed starting PDTs with enthusiasm only to abandon them weeks later, often without formal guidance or follow-up.

Usability and friction encompassed technical, design, and onboarding challenges. These included login issues, confusing interfaces, or difficulty syncing. Posts described how friction at early stages of use sometimes undermined initial motivation, particularly for users less familiar with mobile health technologies. Users frequently reported difficulty launching the app and app crashes with reSET-O and Somryst.

Access and affordability appeared frequently in threads discussing Somryst, Regulora, and SleepioRx. Users cited difficulty obtaining insurance coverage, high out-of-pocket costs, and a lack of clarity around eligibility. In some cases, users reported receiving PDTs through employer benefits or pilot programs but were unsure how to maintain access afterward.

Finally, emotional meaning-making surfaced throughout the dataset. Users often used PDTs as a way to make sense of their conditions, reframe their identities, or regain a sense of control. Whether through narratives of small improvements or critical reflection, posts frequently conveyed emotional labor, hope, frustration, gratitude, and doubt, as integral to the digital therapeutic experience.

## Discussion

This digital ethnographic study offers a nuanced portrayal of the patient and caregiver experience with FDA-cleared PDTs, revealing layers of insight largely absent from clinical trials and formal postmarket surveillance. Reddit, as a spontaneous, culturally diverse forum, served as a window into the lived realities of users navigating these interventions, surfacing themes of ambivalence, emotional engagement, friction, and structural access barriers.

Our findings resonate with prior research documenting the challenges of sustaining engagement with digital behavior change interventions. Perski et al. [22] and Yardley et al. [23] emphasized that low adherence often stems from mismatched user expectations, poor usability, and limited personalization, factors mirrored in our themes of engagement and dropout and usability and friction. Similarly, Torous and Roberts [24] highlighted that mental health apps, despite rigorous development, often fail in real-world deployment due to emotional, contextual, and technical factors that traditional trials overlook. Our data expand on these findings by embedding user narratives within the sociocultural fabric of Reddit communities, revealing that users do not simply abandon PDTs due to inefficacy but also due to emotional fatigue, unmet expectations, and interface friction that undermines initial motivation.

In contrast to studies that rely on surveys or structured interviews, our digital ethnographic approach allowed for observation of naturalistic, user-initiated discussions. This method aligns with Gkotsis et al. [25] who demonstrated Reddit's utility in understanding mental health discourse but builds on it by focusing specifically on regulated therapeutic software rather than general mental health topics. The multidimensionality of user experience captured here, from access and affordability to meaning-making, highlights the inadequacy of current postmarket frameworks that emphasize adverse events and de-identified usage metrics, but neglect the emotional and social dynamics of digital therapeutic use.

Community context significantly influenced user discourse. For example, in r/ADHD, EndeavorRx was evaluated through the lens of parental responsibility and digital literacy, while in r/depression, Rejoyn elicited reflective narratives of emotional vulnerability and therapeutic doubt. These findings underscore the importance of sociotechnical framing: PDTs are not experienced in isolation but within ecosystems of expectation, belief, and support. The same product can be interpreted as empowering or burdensome depending on the community norms and individual identities of its users. This supports the assertion by Eaton et al. [26] that successful digital health interventions must accommodate individual context and evolve dynamically to sustain engagement.

The observed narratives of dropout and disengagement are particularly salient. While randomized controlled trials report average adherence rates and aggregate outcomes, they often exclude users who disengage early or lack digital fluency [26,27]. Reddit provides a counterbalance, capturing the perspectives of users who begin treatment with enthusiasm but gradually withdraw, offering insight into the real-world barriers that shape persistence. This reinforces prior calls for more ecologically valid measures of engagement that reflect emotional, motivational, and technical dimensions [28].

Access and affordability also emerged as critical determinants of therapeutic continuity. Even for FDA-cleared products, users frequently described insurance hurdles, unclear eligibility, or cost-related discontinuation. Our findings further suggest that these access issues may erode user trust and contribute to disengagement, particularly when combined with minimal onboarding support and poor technical usability.

From a regulatory perspective, this study highlights blind spots in current evaluation and surveillance paradigms. While PDTs are reviewed for safety and effectiveness, emotional burden, cognitive fatigue, user trust, and contextual engagement are not routinely assessed, despite being key determinants of therapeutic success in practice. Our results suggest the need for regulators and developers to incorporate social media analysis, or, more broadly, digital ethnography, into postmarket review. This would allow for the real-time identification of usability issues, unmet needs, and sentiment trends, adding a critical patient voice to the regulatory and commercial lifecycle.

Reddit also serves as a decentralized evaluative ecosystem, where users collectively assess and reinterpret therapeutic interventions in real time. These grassroots assessments, while informal, reflect authentic user sentiment and expectations that may diverge significantly from those reported in controlled settings. Listening to these informal narratives is not simply an act of patient engagement; it is essential for refining design, messaging, reimbursement, and onboarding in a rapidly evolving digital therapeutic landscape.

In sum, this study contributes to the growing literature on digital therapeutics by providing an ethnographically grounded account of user experience, situated within real-world online communities. It confirms earlier findings on adherence, trust, and usability while extending them through a cultural and emotional lens. As PDTs continue to expand across therapeutic areas, capturing the lived experience of users will be essential not only for improving outcomes but for ensuring equity, accessibility, and long-term adoption.

Limitations

This study has several limitations that should be acknowledged. First, the sample of Reddit users is not representative of all PDT users due to inherent demographic biases and self-selection bias, as Reddit users may not fully reflect the diversity of patients or caregivers who use PDTs in clinical settings. Additionally, the inability to verify user identities or diagnoses means that the accuracy of the reported experiences is not guaranteed. The findings may also be specific to Reddit's unique platform culture, and the discourse observed may not fully translate to other social media platforms or offline settings. Future studies could mitigate platform-specific biases by triangulating findings across multiple social media platforms to enhance representativeness and thematic robustness. The keyword search strategy, while designed to be comprehensive, may have missed relevant posts that used alternative terminology or slang to discuss PDTs. Moreover, while the 65 posts analyzed provide valuable insights, they may not capture the full spectrum of user experiences across the 11 PDTs included in the study. Further, the asynchronous nature of Reddit posts introduces the risk of misinterpreting user intent or context, as users may express their thoughts in ways that differ from face-to-face communication. Lastly, Reddit's ever-changing user base and content dynamics may limit the replicability of findings in future studies, as community norms and platform algorithms evolve.

## Conclusions

This study demonstrates the value of postmarket digital ethnography in capturing the lived experiences of patients and caregivers using FDA-cleared PDTs. Reddit posts reveal a spectrum of insights, from emotional resonance and dropout patterns to access frustrations and therapeutic meaning-making, that are largely absent from clinical trials or regulatory filings. PDTs exist not just as software applications but as dynamic interventions embedded within cultural, technological, and socioeconomic contexts. To realize their full potential, future evaluation frameworks must look beyond efficacy metrics and embrace the nuanced realities of how people actually engage with digital therapies in everyday life. Social media platforms, when ethically and systematically analyzed, offer a powerful supplement to traditional postmarket surveillance, bringing the patient voice into the heart of digital therapeutic innovation. In the end, incorporating structured digital ethnography into this process could help developers and regulators identify usability barriers, emotional burden, and access gaps in real time.
